# Weight-adjusted waist index and its association with diabetic peripheral neuropathy in patients with type 2 diabetes: a cross-sectional study in coastal Zhejiang province

**DOI:** 10.3389/fnut.2026.1947409

**Published:** 2026-09-21

**Authors:** Liu Axue, Zheng Qidong, Zhuang Hanyao

**Affiliations:** 1 Department of Endocrinology and Metabolism, Health Community Group of Yuhuan Second People’s Hospital, Zhejiang, China; 2 Health Community Group of Yuhuan Second People’s Hospital, Zhejiang, China

**Keywords:** central obesity, cross-sectional study, diabetic peripheral neuropathy, type 2 diabetes mellitus, weight-adjusted waist index

## Abstract

**Background:**

To investigate the association between the weight-adjusted waist index (WWI) and the risk of diabetic peripheral neuropathy (DPN) in patients with type 2 diabetes mellitus (T2DM), and to evaluate its potential as an early screening tool.

**Methods:**

A cross-sectional study was conducted. Patients with T2DM who visited the National Metabolic Management Center (MMC) of Yuhuan Second People’s Hospital between January 2024 and January 2026 were included. Based on sensory nerve conduction velocity results, patients were divided into a DPN group and a non-DPN group. Multivariable logistic regression was used to assess the relationship between WWI and DPN. Subgroup analysis and receiver operating characteristic (ROC) curve analysis were performed to evaluate the discriminative ability of WWI.

**Results:**

A total of 5,646 patients were enrolled, including 1,554 (27.52%) with DPN. Compared with the lowest WWI quartile (Q1), the adjusted odds ratios (ORs) for Q2, Q3, and Q4 were 1.32 (95% CI: 1.08–1.62), 1.91 (95% CI: 1.56–2.33), and 3.64 (95% CI: 2.97–4.46), respectively (*P* for trend < 0.001). ROC curve analysis showed that WWI had a significantly better discriminative ability for DPN than body mass index (BMI). Sex significantly modified the association between WWI and DPN (P for interaction = 0.021), with a stronger discriminative value observed in women.

**Conclusion:**

WWI is an independently associated with for DPN in patients with T2DM. Its screening performance is superior to that of traditional BMI. WWI may serve as a potentially useful tool for DPN screening of DPN.

## Introduction

Diabetic peripheral neuropathy (DPN) is one of the most common chronic microvascular complications of type 2 diabetes mellitus (T2DM). It is characterized by progressive, distal-to-proximal loss of peripheral nerve function, typically presenting with symmetric sensory loss, pain, numbness, and paresthesia in the lower extremities. DPN severely impairs patients’ quality of life and is a major cause of diabetic foot ulcers, amputations, and neuropathic pain (1). Most reports estimate the prevalence of painful DPN (PDPN) among diabetic patients to range from 13 to 35% (2). A comprehensive study conducted in China reported that the prevalence of DPN in the diabetic population was 37% (3). These findings highlight the substantial disease burden of DPN and the urgent need for early screening tools.

Obesity can be classified into generalized obesity and abdominal obesity. Body mass index (BMI) is commonly used to indicate general obesity, but its association with diabetic peripheral neuropathy (DPN) remains controversial. This is partly because BMI does not distinguish between fat mass and lean mass, which may lead to seemingly paradoxical findings regarding the relationship between obesity and neuropathy (4). In contrast, central obesity, characterized by increased waist circumference and visceral fat accumulation, has been consistently identified as an important risk factor for DPN in multiple studies (5). The pathogenic mechanisms involve multiple aspects. Visceral adipose tissue is highly metabolically active and secretes various pro-inflammatory adipokines, such as tumor necrosis factor-alpha (TNF-α) and interleukin-6 (IL-6). These factors can induce systemic chronic low-grade inflammation and insulin resistance, which in turn impair vascular endothelial function, reduce peripheral nerve blood supply, and promote oxidative stress, ultimately leading to axonal degeneration and demyelinating injury of peripheral nerves (6).

A novel obesity indicator proposed in recent years is the weight-adjusted waist index (WWI), calculated as waist circumference (cm) divided by the square root of body weight (kg) (7). WWI is strongly correlated with increased fat mass and decreased muscle mass, and it better reflects abdominal obesity while having a relatively low correlation with confounding factors such as age and sex. Previous studies have shown that WWI is significantly associated with cardiovascular disease, metabolic syndrome, and non-alcoholic fatty liver disease (8–10). Moreover, WWI has been independently associated with mortality in patients with type 2 diabetes (11). However, the relationship between WWI and DPN remains unclear. In particular, evidence is lacking in populations with type 2 diabetes who are at the highest risk for both central obesity and DPN.

## Materials and methods

### Study population

Between January 2024 and January 2026, a total of 6,894 patients with type 2 diabetes mellitus (T2DM) who visited the National Metabolic Management Center (MMC) of Yuhuan Second People’s Hospital were consecutively screened. According to the inclusion and exclusion criteria, a total of 5,646 patients were ultimately enrolled in this study. The detailed participant selection process is illustrated in Figure 1.

**FIGURE 1:**
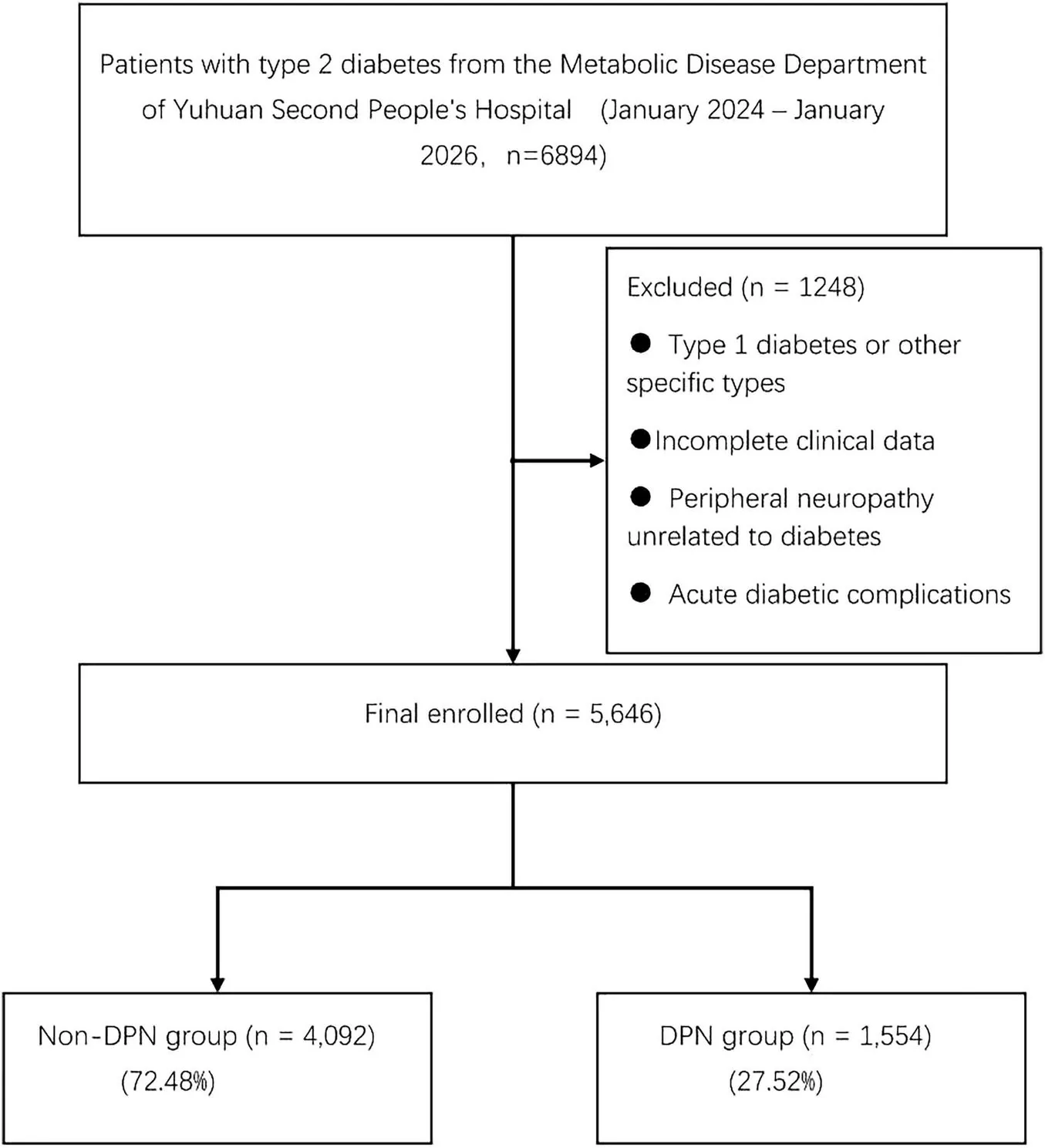
Flowchart of participant selection.

The inclusion criteria were as follows: (1) diagnosis of diabetes according to the American Diabetes Association criteria established in 2022; (2) complete clinical data.

The exclusion criteria were as follows: (1) type 1 diabetes, other specific types of diabetes, or secondary diabetes; (2) peripheral neuropathy unrelated to diabetes; and (3) acute diabetic complications.

### Sample size calculation

The sample size was calculated based on the formula for cross-sectional studies:


$$n={\left(Z\alpha /2\right)}^{2}\times \text{P}\times {\text{(1-P)/d}}^{\text{2}},$$

where Zα/2 = 1.96 (for a two-sided significance level of α = 0.05), *P* = 0.30 [estimated prevalence of DPN based on prior literature (2)], and *d* = 0.03 (margin of error). The minimum required sample size was calculated to be 896 participants. The final sample size of 5,646 participants in this study exceeded this requirement, providing sufficient statistical power for the analyses (12).

### Data collection

Patient data were collected through standardized questionnaires and electronic medical records. The collected variables included age, sex, height, weight, and waist circumference. Biochemical parameters included glycated hemoglobin (HbA1c), total cholesterol (TC), high-density lipoprotein cholesterol (HDL-c), low-density lipoprotein cholesterol (LDL-c), triglycerides (TG), alanine aminotransferase (ALT), and aspartate aminotransferase (AST). Information on medical history, including hypertension, coronary heart disease, and stroke, was also recorded.

WWI was calculated as waist circumference (cm) divided by the square root of body weight (kg). BMI was calculated as weight (kg) divided by height squared (m^2^). Visceral fat area was measured using bioelectrical impedance analysis (HDS-2000 body composition analyzer). All measurements were performed by trained medical professionals to ensure reliability.

### Diagnosis of DPN

DPN was defined as the presence of symptoms and/or signs of peripheral nerve dysfunction in patients with diabetes after the exclusion of other causes (13).

QST is a technique used to assess sensory neuropathy. It detects both small and large fiber neuropathy by applying standardized and quantified sensory stimuli, such as touch, vibration, and temperature. Previous studies have reported that the vibration perception threshold (VPT) has a sensitivity of 85.19% and a specificity of 88.68% for diagnosing DPN, suggesting its utility for early DPN screening (14).

In this study, VPT was measured at the base of the great toe or the dorsum of the foot using a digital biothesiometer (sensiometer). Measurements were repeated three times, and the average value was automatically calculated. DPN was diagnosed if the VPT in any tested location exceeded the normal threshold. All measurements were performed by trained nurses from the MMC. All devices were calibrated before use and on a regular basis to ensure measurement accuracy and reliability.

### Statistical analysis

All statistical analyses were performed using R software (version 4.2.0). Continuous variables are presented as mean ± standard deviation (x̄ ± s). Group comparisons were performed using the independent samples *t*-test or the Mann–Whitney U test, as appropriate. Categorical variables are presented as numbers (percentages) and were compared using the chi-square test or Fisher’s exact test. WWI was categorized into quartiles (Q1 to Q4).

Multivariable logistic regression analysis was used to assess the association between WWI and DPN. Three models were developed to adjust for potential confounders:

1. Model 1: unadjusted.
2. Model 2: adjusted for sex.
3. Model 3: adjusted for sex, history of hypertension, coronary heart disease, stroke, BMI, visceral fat area, HbA1c, ALT, AST, TC, and HDL-c.

Restricted cubic spline (RCS) analysis with four knots was performed to explore the nonlinear relationship between WWI and DPN. Subgroup analyses were conducted by categorizing WWI into quartiles (Q1–Q4). Stratification was performed according to sex, history of hypertension, coronary heart disease, and stroke. The P value for interaction was calculated.

Receiver operating characteristic (ROC) curves were used to evaluate the predictive performance of WWI and BMI for DPN. The area under the curve (AUC) was calculated. The DeLong test was used to compare the AUCs of WWI and BMI. A two-sided *P* value < 0.05 was considered statistically significant.

## Results

### Baseline characteristics of the study population

As shown in Table 1, a total of 5,646 patients with type 2 diabetes were included in this study. Among them, 1,554 (27.52%) were diagnosed with diabetic peripheral neuropathy (DPN), and the remaining 4,092 (72.48%) were classified as the non-DPN group.

**TABLE 1:** Baseline characteristics of the study population.

Variables	Total (*n* = 5646)	Non-DPN (*n* = 4092)	DPN (*n* = 1554)	Statistic	*P*
BMI, Mean ± SD	25.85 ± 4.01	26.13 ± 4.10	25.10 ± 3.64	*t* = 9.13	**< 0.001**
VAT, Mean ± SD	106.02 ± 42.83	106.09 ± 42.51	105.81 ± 43.68	*t* = 0.22	0.826
HbA1c, Mean ± SD	0.07 ± 0.02	0.07 ± 0.02	0.08 ± 0.02	*t* = −4.60	**< 0.001**
ALT, Mean ± SD	35.40 ± 50.27	37.44 ± 54.65	30.03 ± 35.71	*t* = 5.96	**< 0.001**
AST, Mean ± SD	29.93 ± 38.21	29.70 ± 36.82	30.54 ± 41.65	*t* = −0.73	0.463
TC, Mean ± SD	5.09 ± 1.30	5.21 ± 1.29	4.79 ± 1.30	*t* = 10.78	**< 0.001**
HDL-c, Mean ± SD	1.19 ± 0.37	1.19 ± 0.36	1.17 ± 0.39	*t* = 1.60	0.109
Sex, *n* (%)				χ^2^ = 0.84	0.360
Female	2285 (40.47)	1641 (40.10)	644 (41.44)		
Male	3361 (59.53)	2451 (59.90)	910 (58.56)		
Hypertension, *n* (%)				χ^2^ = 197.90	**< 0.001**
No	3140 (55.69)	2510 (61.43)	630 (40.59)		
Yes	2498 (44.31)	1576 (38.57)	922 (59.41)		
CHD, *n* (%)				χ^2^ = 120.71	**< 0.001**
No	5090 (90.28)	3798 (92.95)	1292 (83.25)		
Yes	548 (9.72)	288 (7.05)	260 (16.75)		
Stroke, *n* (%)				χ^2^ = 75.44	**< 0.001**
No	5500 (97.55)	4031 (98.65)	1469 (94.65)		
Yes	138 (2.45)	55 (1.35)	83 (5.35)		
WWI quantile, *n* (%)				χ^2^ = 316.86	**< 0.001**
Q1	1409 (24.99)	1178 (28.82)	231 (14.88)		
Q2	1410 (25.00)	1115 (27.28)	295 (19.01)		
Q3	1410 (25.00)	1008 (24.66)	402 (25.90)		
Q4	1410 (25.00)	786 (19.23)	624 (40.21)		

ALT, alanine aminotransferase; AST, aspartate aminotransferase; BMI, body mass index; DPN, diabetic peripheral neuropathy; HbA1c, glycated hemoglobin; HDL-c, high-density lipoprotein cholesterol; SD, standard deviation; TC, total cholesterol; VAT, visceral fat area; WWI, weight-adjusted waist index. *Bold values indicate statistical significance (*p* < 0.05).

Compared with the non-DPN group, patients in the DPN group had significantly lower BMI, ALT, and TC levels (all *P* < 0.001). In contrast, HbA1c levels were significantly higher in the DPN group (*P* < 0.001). The rates of hypertension history (59.41% vs. 38.57%), coronary heart disease (16.75% vs. 7.05%), and stroke (5.35% vs. 1.35%) were significantly higher in the DPN group than in the non-DPN group, and all differences were statistically significant [all (*P* < 0.001); Table 1]. No significant differences were observed between the two groups in visceral fat area, AST, HDL-c, or sex distribution (all *P* > 0.05).

The distribution of WWI quartiles differed significantly between the two groups (*P* < 0.001). As the WWI quartile increased from Q1 to Q4, the proportion of patients in the DPN group gradually increased.

### Multivariable logistic regression analysis

As shown in Table 2, the single-factor logistic regression analysis indicates, compared with the lowest quartile of WWI (Q1), the odds ratios (ORs) for Q2, Q3, and Q4 were 1.35 (95% CI: 1.11–1.63), 2.03 (95% CI: 1.69–2.44), and 4.05 (95% CI: 3.40–4.83), respectively (all *P* < 0.05).

**TABLE 2:** Univariable and multivariable logistic regression analysis of factors associated with DPN.

Variables	Univariate analysis					Multivariate analysis				
	β	S.E	Z	*P*	OR (95%CI)	β	S.E	Z	*P*	OR (95%CI)
WWI quantile										
Q1				1.00 (Reference)				1.00 (Reference)		
Q2	0.30	0.10	3.08	**0.002**	1.35 (1.11 ∼ 1.63)	0.28	0.10	2.71	**0.007**	1.32 (1.08 ∼ 1.62)
Q3	0.71	0.09	7.63	**< 0.001**	2.03 (1.69 ∼ 2.44)	0.65	0.10	6.34	**< 0.001**	1.91 (1.56 ∼ 2.33)
Q4	1.40	0.09	15.58	**< 0.001**	4.05 (3.40 ∼ 4.83)	1.29	0.10	12.49	**< 0.001**	3.64 (2.97 ∼ 4.46)
Sex (male vs. female)										
Female				1.00 (Reference)				1.00 (Reference)		
Male	−0.06	0.06	−0.92	0.360	0.95 (0.84 ∼ 1.07)	0.10	0.07	1.35	0.178	1.10 (0.96 ∼ 1.27)
Hypertension (yes vs. no)										
No				1.00 (Reference)				1.00 (Reference)		
Yes	0.85	0.06	13.90	**< 0.001**	2.33 (2.07 ∼ 2.63)	0.69	0.07	10.19	**< 0.001**	2.00 (1.75 ∼ 2.28)
CHD (yes vs. no)										
No				1.00 (Reference)				1.00 (Reference)		
Yes	0.98	0.09	10.68	**< 0.001**	2.65 (2.22 ∼ 3.17)	0.59	0.11	5.46	**< 0.001**	1.80 (1.46 ∼ 2.23)
Stroke (yes vs. no)										
No				1.00 (Reference)				1.00 (Reference)		
Yes	1.42	0.18	8.05	**< 0.001**	4.14 (2.93 ∼ 5.85)	0.80	0.19	4.17	**< 0.001**	2.22 (1.52 ∼ 3.23)
BMI	−0.07	0.01	−8.54	**< 0.001**	0.93 (0.92 ∼ 0.95)	−0.11	0.01	−9.05	**< 0.001**	0.89 (0.87 ∼ 0.91)
Visceral fat area	−0.00	0.00	−0.22	0.826	1.00 (1.00 ∼ 1.00)	0.01	0.00	2.93	**0.003**	1.01 (1.01 ∼ 1.01)
HbA1c	5.80	1.27	4.58	**< 0.001**	1.06 (1.03 ∼ 1.08)	8.62	1.46	5.92	**< 0.001**	1.09 (1.06 ∼ 1.12)
ALT	−0.01	0.00	−6.19	**< 0.001**	0.99 (0.99 ∼ 0.99)	−0.01	0.00	−4.04	**< 0.001**	0.99 (0.99 ∼ 0.99)
AST	0.00	0.00	0.73	0.465	1.00 (1.00 ∼ 1.00)	0.01	0.00	2.44	**0.015**	1.01 (1.01 ∼ 1.01)
TC	−0.27	0.03	−10.64	**< 0.001**	0.76 (0.72 ∼ 0.80)	−0.22	0.03	−7.65	**< 0.001**	0.81 (0.76 ∼ 0.85)
HDL-c	−0.14	0.08	−1.67	0.094	0.87 (0.74 ∼ 1.02)	0.13	0.10	1.31	0.189	1.13 (0.94 ∼ 1.37)

ALT, alanine aminotransferase; AST, aspartate aminotransferase; BMI, body mass index; CHD, coronary heart disease; CI, confidence interval; DPN, diabetic peripheral neuropathy; HbA1c, glycated hemoglobin; HDL-c, high-density lipoprotein cholesterol; OR, odds ratio; SE, standard error; TC, total cholesterol; WWI, weight-adjusted waist index. Multivariable model adjusted for sex, hypertension, coronary heart disease, stroke, BMI, visceral fat area, HbA1c, ALT, AST, TC, and HDL-c. *Bold values indicate statistical significance (*p* < 0.05).

After adjusting for sex, history of hypertension, coronary heart disease, stroke, BMI, visceral fat area, HbA1c, ALT, AST, TC, and HDL-c, WWI remained an independent risk factor for DPN. Compared with Q1, the adjusted ORs for Q2, Q3, and Q4 were 1.32 (95% CI: 1.08–1.62), 1.91 (95% CI: 1.56–2.33), and 3.64 (95% CI: 2.97–4.46), respectively. The ORs showed a significant increasing trend across quartiles (P for trend < 0.001).

### Sensitivity analysis using multiple models

Table 3 presents the results of the stepwise adjustment models. In Model 1 (unadjusted), the OR for Q4 versus Q1 was 4.05 (95% CI: 3.40–4.83). In Model 2 (adjusted for sex), the OR was 4.20 (95% CI: 3.51–5.02). In Model 3 (fully adjusted), the OR was 3.64 (95% CI: 2.97–4.46). Across all three models, the ORs for WWI quartiles showed a consistent increasing trend (all *P* for trend < 0.001). These findings indicate that the association between WWI and DPN is robust.

**TABLE 3:** Multivariable logistic regression analysis of the association between WWI and DPN across three models.

Variables	Model1		Model2		Model3	
	OR (95%CI)	*P*	OR (95%CI)	*P*	OR (95%CI)	*P*
WWI quantile						
Q1	1.00 (Reference)		1.00 (Reference)		1.00 (Reference)	
Q2	1.35 (1.11 ∼ 1.63)	**0.002**	1.34 (1.10 ∼ 1.62)	**0.003**	1.32 (1.08 ∼ 1.62)	**0.007**
Q3	2.03 (1.69 ∼ 2.44)	**< 0.001**	2.04 (1.70 ∼ 2.45)	**< 0.001**	1.91 (1.56 ∼ 2.33)	**< 0.001**
Q4	4.05 (3.40 ∼ 4.83)	**< 0.001**	4.20 (3.51 ∼ 5.02)	**< 0.001**	3.64 (2.97 ∼ 4.46)	**< 0.001**

CI, confidence interval; DPN, diabetic peripheral neuropathy; OR, odds ratio; WWI, weight-adjusted waist index. Model 1: Crude model (unadjusted). Model 2: Adjusted for sex. Model 3: Adjusted for sex, history of hypertension, coronary heart disease, stroke, BMI, visceral fat area, HbA1c, ALT, AST, TC, and HDL-c. *Bold values indicate statistical significance (*p* < 0.05).

### Restricted cubic spline analysis

Restricted cubic spline (RCS) analysis was performed to examine the dose-response relationship between WWI and DPN. As shown in Figure 2, the RCS results revealed a non-linear positive association between WWI and the risk of DPN [(*P* < 0.001)]).

**FIGURE 2:**
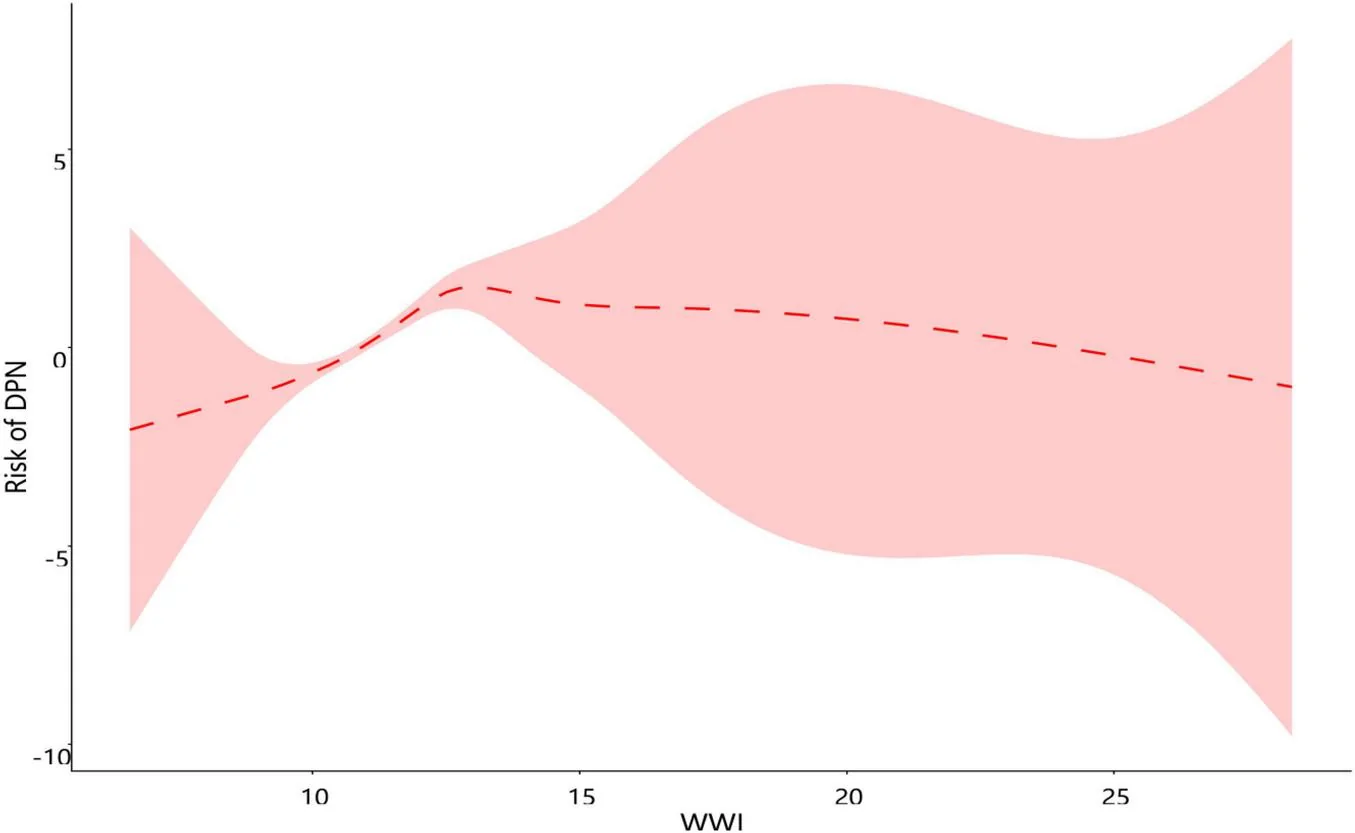
Restricted cubic spline (RCS) analysis of the association between WWI and DPN risk.

### Subgroup analysis

Subgroup analysis (Figure 3) shows the odds ratios (ORs) and 95% confidence intervals (CIs) for the highest versus lowest quartile of WWI, stratified by sex, hypertension, coronary heart disease, and stroke history. The overall OR is indicated by the dashed vertical line. The P value for interaction was 0.021 for sex, 0.327 for hypertension, 0.208 for coronary heart disease, and 0.499 for stroke, indicating that sex significantly modified the association between WWI and DPN, with a stronger association observed in female patients.

**FIGURE 3:**
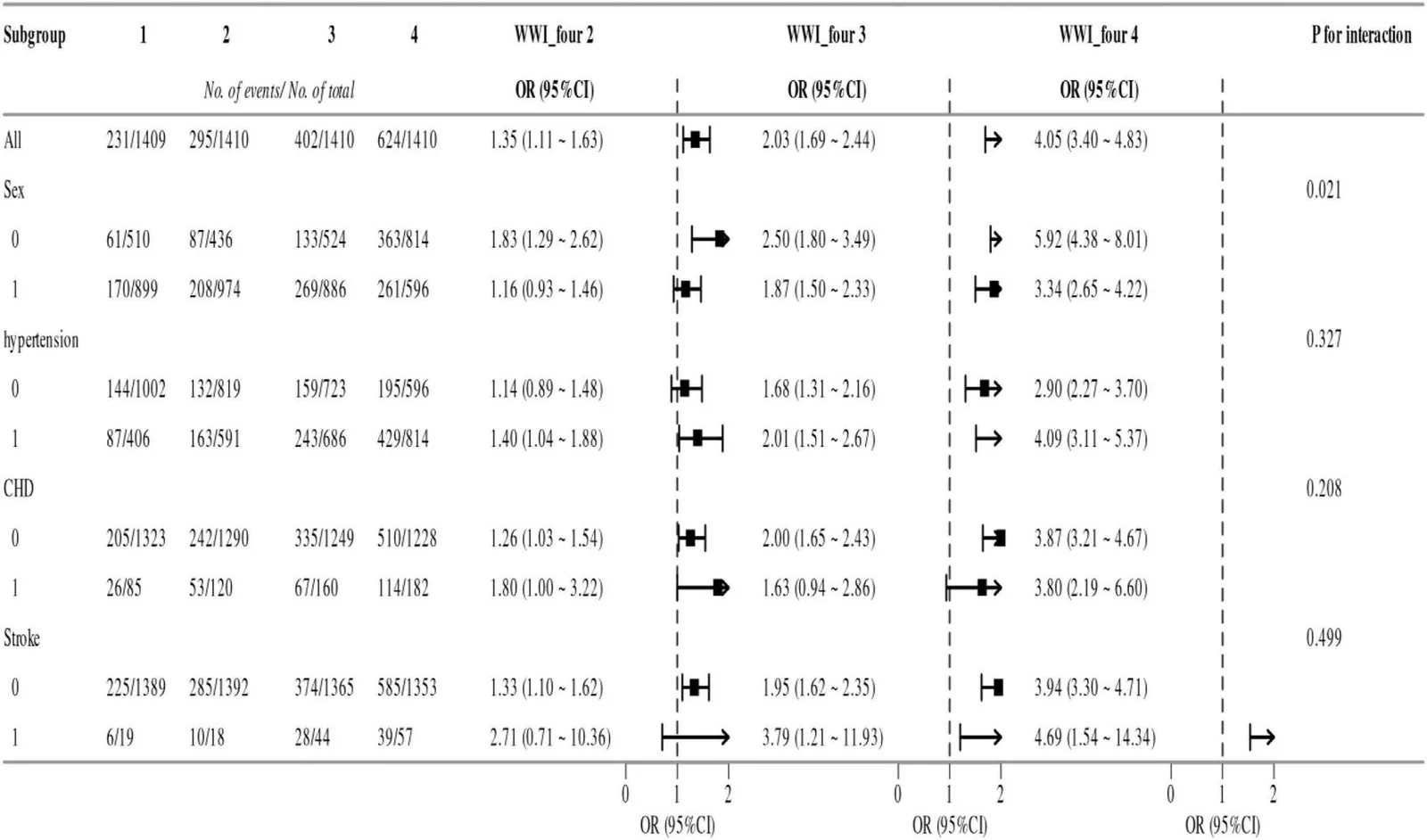
Subgroup analysis of the association between WWI and DPN. For categorical variables: Sex (0 = female, 1 = male); Hypertension (0 = no, 1 = yes); CHD (0 = no, 1 = yes); Stroke (0 = no, 1 = yes).

### Screening performance of WWI for DPN

Receiver operating characteristic (ROC) curve analysis was performed (Figure 4). The AUC of WWI for predicting DPN was 0.654 (95% CI: 0.634–0.673), which was significantly higher than that of BMI (AUC = 0.563, 95% CI: 0.543–0.582). The DeLong test showed that the difference between the two AUCs was statistically significant (*P* < 0.001). These results suggest that WWI is superior to traditional BMI in screening for DPN.

**FIGURE 4:**
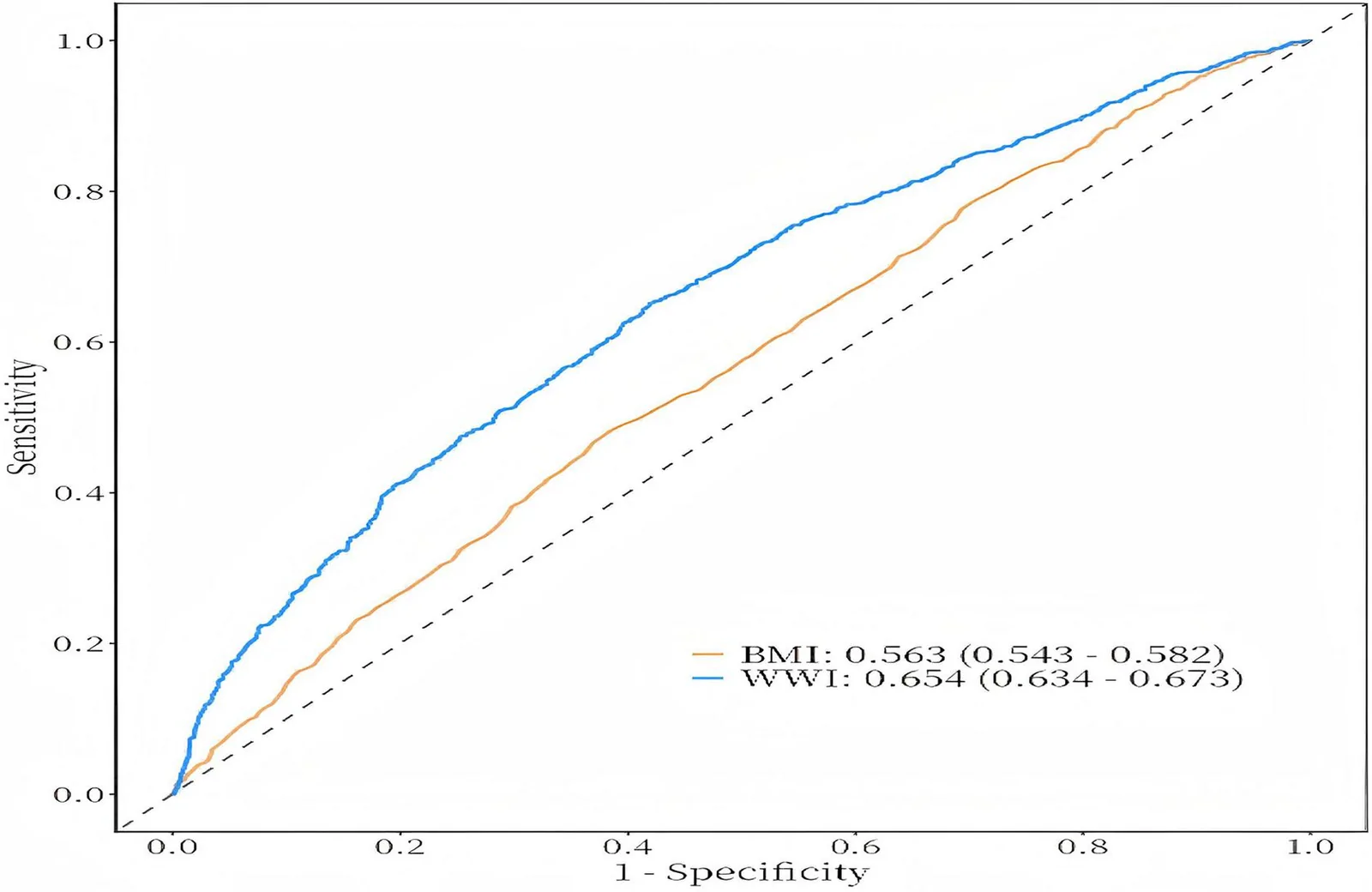
Receiver operating characteristic (ROC) curves of WWI and BMI for predicting DPN.

## Discussion

This study was based on a cross-sectional survey of 5,646 patients with T2DM from the coastal region of Zhejiang Province, China. For the first time, we systematically investigated the relationship between WWI, a novel obesity indicator, and the risk of DPN. The main findings are as follows. First, higher WWI quartiles were independently associated with an increased risk of DPN in a dose-response manner. Second, WWI showed a superior screening performance for DPN compared with traditional BMI. Third, sex significantly modified the association between WWI and DPN.

### Independent association between WWI and DPN

The core finding of this study is that, even after comprehensive adjustment for known risk factors of DPN–including BMI, visceral fat area, HbA1c, and history of cardiovascular and cerebrovascular diseases–WWI quartiles remained significantly and positively associated with DPN risk. The effect size was substantial (Q4 vs. Q1: OR = 3.64). This result is consistent with previous studies reporting that central obesity is significantly associated with neuropathy (15).

Central obesity has been shown to promote the development of DPN through multiple mechanisms. Visceral adipose tissue releases various adipocytokines, such as tumor necrosis factor-alpha (TNF-α) and interleukin-6 (IL-6). These factors trigger systemic chronic low-grade inflammation and insulin resistance, thereby exacerbating the pathological process of diabetes (16).

Of note, in the present study, BMI appeared as a protective factor for DPN in both univariable and multivariable analyses (OR = 0.89). However, a prospective cohort study from the UK Biobank found that higher BMI was associated with an increased risk of DPN (17). Similarly, a study involving adolescents and young adults also suggested that higher BMI was related to a higher risk of DPN (18). These findings are consistent with the “obesity paradox” reported in some studies.

One possible explanation for the obesity paradox is that using traditional BMI as an obesity indicator may overlook individuals with abdominal obesity. In contrast, WWI corrects for body weight and can more specifically reflect abdominal fat accumulation, thereby overcoming the limitations of BMI. Therefore, WWI may be a more suitable indicator than BMI for predicting the risk of DPN.

### WWI versus BMI: central obesity indicators for DPN in T2DM patients

In this study, we found that WWI was independently and positively associated with DPN in patients with T2DM, with a dose-response relationship. Importantly, WWI demonstrated better discriminative ability than BMI in identifying DPN, suggesting that WWI may serve as a more effective anthropometric indicator for DPN risk assessment.

Interestingly, in the present study, BMI appeared as a protective factor for DPN in the multivariable analysis (OR = 0.89, 95% CI: 0.87–0.91). This seemingly paradoxical finding may be explained by the “obesity paradox” phenomenon observed in this populations (19).

A possible explanation lies in the influence of sarcopenic obesity, a condition characterized by reduced muscle mass, decreased muscle strength, and fat accumulation. The diagnosis of sarcopenic obesity typically requires simultaneous assessment of muscle mass, muscle strength, and body fat percentage (20). Of note, sarcopenia often develops in the early stages of disease (21), rather than being merely a passive consequence of aging. For example, studies have shown that the decline in physical function among middle-aged individuals is more closely associated with a sedentary lifestyle than with biological aging (22). Even in healthy young adults, 10 days of bed rest can lead to approximately 5% loss of muscle mass and a 10% reduction in muscle strength. Furthermore, chronic diseases, chronic inflammation, and unhealthy lifestyle factors can all contribute to the development and progression of sarcopenia, which is also more prevalent in patients with chronic diseases (23).

Based on the above mechanisms, some patients with T2DM may have concurrent central obesity and reduced muscle mass, while their BMI remains within the normal range or even at a lower level. In this context, BMI may not accurately reflect the actual degree of fat accumulation, and may even mask the increased risk of DPN associated with central obesity.

A prospective cohort study in the United States reported a J-shaped association between BMI and all-cause mortality in men. After excluding individuals with low lean mass, the J-shaped association became linear, suggesting that differences in body fat and muscle mass can influence the relationship between BMI and disease outcomes (24). A 2014 Chinese study indicated that lower BMI may be a novel independent risk factor for DPN (25). Furthermore, a systematic review and meta-analysis of 97 prospective studies reported that, compared with normal weight (BMI 18.5–24.9), obesity (BMI ≥ 30) was associated with higher all-cause mortality, whereas overweight (BMI 25–29.9) was associated with lower all-cause mortality (26).

However, this does not imply that obesity is beneficial. Rather, it highlights the limitations of BMI as an obesity measure in populations. In light of these considerations, As a novel obesity index (27), WWI differs from traditional anthropometric measures by providing a more detailed assessment of body composition, as it is positively correlated with abdominal fat and negatively correlated with muscle mass. The consistent positive association between WWI and DPN observed in the present study suggests that WWI may be a more suitable indicator for assessing DPN risk in this population.

### Sex differences

This study found that sex significantly modified the association between WWI and DPN (*P* for interaction = 0.021). The effect of WWI on DPN was larger in women. This result is consistent with previously reported sex-related differences in DPN. A study investigating risk factors for peripheral artery disease and DPN in patients with type 2 diabetes identified female sex as an independent risk factor for DPN (28).

In addition, hormonal changes occur with aging in women. After menopause, the ratio of androgens (i.e., testosterone) to estradiol increases. This leads to a shift in fat distribution from a peripheral to a central pattern. Therefore, the same waist circumference may correspond to greater visceral fat accumulation and more severe metabolic disturbances in postmenopausal women (29).

Furthermore, female patients with DPN may be more sensitive to neuropathic symptoms such as pain and numbness. Abraham et al. reported that women with neuropathy experience more intense pain than men (30). This may contribute to a higher likelihood of DPN diagnosis in women. However, the mechanisms underlying sex differences in chronic pain are not fully understood. Sex hormones are known to contribute to sexual differentiation of the nervous system and are hypothesized to be involved in pain modulation (31). Fluctuations in estrogen levels may increase pain sensitivity in women, whereas testosterone in men may promote pain relief. Sex hormones may also interact with the neuroimmune system and alter the activity of sensory neurons.

From a clinical perspective, this sex difference suggests that future DPN screening strategies should pay particular attention to central obesity in female patients.

### Strengths and limitations

This study has several notable strengths. First, to our knowledge, it is the first study to systematically investigate the association between WWI and DPN in a population with T2DM from coastal Chinese cities, providing an innovative Contribution To The Field. Second, the relatively large sample size (*n* = 5,646) and the use of standardized data from the Metabolic Management Center (MMC) ensured high data quality. Third, we adjusted for a comprehensive set of known confounders and employed multiple modeling strategies together with subgroup analyses to evaluate the robustness of our findings. Fourth, HbA1c levels were standardized to clinically interpretable units.

In addition, several limitations of this study should be acknowledged. First, the cross-sectional study design precludes the determination of causality. Future prospective cohort studies are warranted to validate our findings. Second, all participants were recruited from a single hospital in the coastal region of Zhejiang Province, China, which may introduce selection bias and limit the generalizability of our findings to other populations such as those from inland regions. Third, the AUC of WWI for predicting DPN was 0.654, indicating its limited predictive performance as a single screening tool. Fourth, the diagnosis of DPN in this study was based solely on VPT measurement, which mainly assesses the function of large nerve fibers and may not fully capture small fiber neuropathy. This may lead to an underestimation of the prevalence of DPN. Fifth, we did not include potential confounders that may affect nerve function, such as vitamin B12 levels and thyroid function. Therefore, residual confounding may exist.

Future multicenter, prospective studies are needed to further validate the predictive ability of WWI for the development of DPN. In addition, combining WWI with other screening indicators to establish an optimal screening model is worth exploring.

## Conclusion

This cross-sectional study demonstrates that WWI is independently and positively associated with DPN in patients with T2DM from the coastal region of Zhejiang Province, China, with a dose-response relationship. WWI showed better discriminative ability than traditional BMI in identifying DPN, particularly in female patients. As a simple, non-invasive, and easily obtainable novel indicator of central obesity, WWI may serve as a useful reference for DPN screening in clinical practice. Future prospective studies are needed to confirm the causal relationship between WWI and DPN.

## Data Availability

The raw data supporting the conclusions of this article will be made available by the authors, without undue reservation.
